# Association between perceived exposure to secondhand smoke and depression independent of biomarker-measured exposure

**DOI:** 10.1186/s12889-025-23967-8

**Published:** 2025-08-25

**Authors:** Dongkyu Lee, Hyeon Chang Kim, Young-Chul Jung, Sun Jae Jung

**Affiliations:** 1 Department of Preventive Medicine, Yonsei University College of Medicine, Seoul, Korea; 2 Department of Public Health, Yonsei University Graduate School, Seoul, Korea; 3 Department of Psychiatry, Yonsei University College of Medicine, Seoul, Korea; 4 Institute for Innovation in Digital Healthcare, Yonsei University, Seoul, Korea

**Keywords:** Tobacco smoke pollution, Secondhand smoke, Depression, Perception, Biomarker

## Abstract

**Background:**

Perceived exposure to secondhand smoke has previously not been distinguished from actual exposure dose when considering the association with depression. This cross-sectional study evaluated whether perceived exposure to secondhand smoke was associated with depression after adjusting for biomarker-based exposure.

**Methods:**

Adult non-smokers and ex-smokers (*N* = 16,926) were sampled from the Korea National Health and Nutrition Examination Survey from 2014 to 2020 biennially. Perceived exposure was defined by self-reported indoor secondhand smoke exposure in workplaces, households, or public locations in the past 7 days. Urine cotinine was used as the biomarker-measured exposure to secondhand smoke. Depression was defined as scoring 10 or above on the Patient Health Questionnaire-9. Logistic regression evaluated the association between perceived exposure and depression while adjusting for biomarker-based exposure, demographics, socioeconomic status, and comorbidities.

**Results:**

Perceived exposure to secondhand smoke was associated with depression (adjusted odds ratio [aOR]: 1.60, 95% confidence interval [CI]: 1.31–1.95). Perceived exposure in occupational (aOR: 1.62, 95% CI: 1.17–2.25), household (aOR: 1.56, 95% CI: 1.14–2.13), and public (aOR: 1.57, 95% CI: 1.28–1.93) settings showed similar strengths of association with depression. Perceived exposure in one location (aOR: 1.49, 95% CI: 1.20–1.85) to three locations (aOR: 3.06, 95% CI: 1.55–6.07) showed dose–response associations with depression.

**Conclusions:**

Perceived exposure to secondhand smoke was associated with depression independent of actual biological exposure. Creating comprehensive smokefree environments should be prioritized to protect the general population from depression, with additional measures to reduce sensory cues of secondhand smoke where complete bans are not yet feasible.

**Supplementary Information:**

The online version contains supplementary material available at 10.1186/s12889-025-23967-8.

## Background

Secondhand smoke (SHS) is a public health risk factor that causes involuntary damage to non-smokers through unfiltered substances [1, 2]. The current National Health Promotion Act of Korea, article 9, bans smoking within almost all indoor facilities [3]. Only a few exceptions exist, such as individual households and workplaces smaller than 1,000 m^2^. Meanwhile, the proportion of adults who reported experiencing SHS within a week is in a declining trend. From 2014 to 2020, the proportions have been reduced within workplaces (40.7% to 10.7%), households (10.3% to 3.8%), and public locations (49.6% to 10.5%) [4]. However, surveillance on violations of the National Health Promotion Act is not very effective [5], and comprehensive nationwide statistics for Korea are still limited. Only some districts provide summary statistics. For example, one district within the capital city Seoul reported that 14,275 violations were fined [6], from a population of approximately 400,000 [7] and a smoking rate of 11.4% [8] in 2023.

Not only does SHS cause physical illnesses, which add up to a burden of 36 million disability-adjusted life years per year [9], but it also shows associations with psychiatric illnesses. Studies have found SHS to be associated with mental illnesses throughout stages of life, such as attention-deficit/hyperactivity disorder in children [10], depression in adolescents and adults [11–13], and cognitive decline in elderly populations [14]. Among the mental illnesses associated with SHS, depression is the leading cause of disease burden, accounting for 1.8% of the total burden in 2019 [15].

However, previous studies have solely defined SHS exposure either by perception (i.e., self-reported exposure) or by biomarkers of tobacco smoke [12, 13]. Additionally, heterogeneity across results is observed. While significant associations between perceived SHS exposure and depression were found [13], studies using biomarker-measured SHS showed weaker [13] or nonsignificant [16] associations with depression. This discrepancy suggests that perceived exposure to SHS could be a distinct risk factor for depression independent of actual exposure to SHS. In addition, SHS exposure is perceived as irritating, annoying, and harmful [17–19], making exposure to SHS an aversive and stressful experience that may contribute to depression. Therefore, this study investigated the association between perceived SHS exposure and depression, adjusting for biomarker-based exposure dose in non-smokers and ex-smokers, since no prior study has simultaneously assessed perceived exposure to SHS and the biological degree of exposure to SHS in relationship with depression.

## Methods

### Data source

The Korea National Health and Nutrition Examination Survey (KNHANES) is a national cross-sectional survey conducted yearly by the Korea Disease Control and Prevention Agency [20]. The survey evaluates the general health of the population, monitors chronic disease prevalence, and incorporates physical examinations and laboratory test results. Interviewing, physical examinations, and obtainment of laboratory samples were performed by trained interviewers and medical staff [20]. The KNHANES includes non-institutionalized citizens of South Korea, aged 1 year and older, as participants. A multi-stage clustered probability design was used to sample the participants, enabling representation of the Korean population while maintaining a large number of participants [20].

### Participant selection

The KNHANES data from the years 2014, 2016, 2018, and 2020 were used. Of 19,046 non-smoker and ex-smoker adults, participants with incomplete reporting on perceived SHS exposure (exposure variable, *N* = 6), incomplete reporting on depressive symptoms (outcome variable, *N* = 1,100), and missing data on cotinine (SHS biomarker covariate, *N* = 860) were excluded from the analytic domain, leaving 17,080 participants. Then, participants with missing information on income (*N* = 48), education (*N* = 9), type of occupation (*N* = 19), and body mass index (*N* = 78) were excluded, leaving 16,926 participants as the final analytic domain.

### Exposure (perceived secondhand smoke exposure)

Perceived SHS exposure was defined based on self-reported exposure. The participants were asked three questions:"In the past 7 days, were you exposed to tobacco smoke indoors from another person in your 1) workplace? 2) household? 3) public places except for designated smoking areas?" [21]. Participants who did not experience SHS in any of the locations above were classified as the reference group, while participants with perceived SHS exposure in any of these locations were classified as the exposed group.

### Outcome (depression)

Depression was measured using the Patient Health Questionnaire-9 (PHQ-9) [22], which has been validated in the Korean population [23]. The questionnaire consists of 9 items corresponding to the 9 items in criteria A for major depressive disorder in the Diagnostic and Statistical Manual of Mental Disorders [22]. Each item of the PHQ-9 can be answered with a score of 0 (not at all), 1 (several days), 2 (more than one week), and 3 (nearly every day). Participants with total scores of 10 or above were classified as depressed, while those scoring below 10 were set as the reference group.

### Main covariate (biomarker-measured secondhand smoke exposure)

Biological exposure dose to SHS was measured with urine cotinine, a metabolite of nicotine [24]. Urine cotinine was measured once, during a single visit to the mobile examination center [20], immediately before other health examinations were performed and questionnaires were administered [25]. Cotinine (ng/mL) measurements with values under the limit of detection (LOD) were substituted with LOD/$\sqrt{2}$ [26]. The LOD was 0.27399 ng/mL in years 2014 to 2018, and 0.5 ng/mL in 2020 [27]. Finally, cotinine values were log-transformed for normalization, in line with previous studies [28–30].

### Other covariates

Demographic factors (age, sex), socioeconomic factors (income, education, marital status, number of cohabitants, and occupation type), and comorbidities (metabolic, cardiovascular, neurovascular, thyroid, and neoplastic) were included as covariates. Details on covariate definitions are provided in eMethods 1.

### Statistical analysis

Characteristics of the study population were compared between depressed and reference groups using chi-square tests for categorical variables and t-tests for continuous variables. Logistic regression (PROC SURVEYLOGISTIC) was performed to evaluate the association between perceived SHS exposure and depression while adjusting for log-transformed cotinine and other covariates. Odds ratios (ORs) and Wald confidence intervals (CIs) were calculated. All subsequent analyses also adjusted for cotinine and other covariates.

Several analyses were conducted to further evaluate the association between perceived SHS and depression. First, interaction between perceived and biomarker-based SHS was tested using the likelihood ratio test. Additionally, perceived exposure from individual locations was used as the exposure variable to evaluate whether the associations between perceived exposure from individual locations and depression were consistent across locations, and not driven by a single location. Then, the total number of locations (i.e., workplace/household/public place) where an individual reported perceived SHS exposure was used as the exposure variable to assess the dose–response relationship between perceived exposure to SHS and depression. Finally, effect heterogeneity analysis was conducted on an exploratory basis, with details provided in eMethods 2.

Several sensitivity analyses were conducted. First, categorical age and number of cohabitants were used instead of continuous variables to account for potential information loss due to censoring of continuous variables. Then, the analysis was restricted to participants without prior physician-diagnosed depression to reduce the possibility of reverse causality. In addition, 4-(methylnitrosamino)−1-(3-pyridyl)−1-butanol (NNAL) [31], an alternative biomarker of SHS measured in random subsamples of the KNHANES participants in 2016 and 2018, was used instead of cotinine. This biomarker, with a longer half-life of 10–16 days [31], was used to account for a wider detection window than cotinine [32], as cotinine can detect only recent biological exposure to SHS due to its short half-life of approximately 15 h [33]. Furthermore, the analysis was restricted to biomarker-confirmed non-smokers and ex-smokers by filtering out hidden smokers. Alternative outcome definitions were also tested, such as different cut-off scores for the PHQ-9 or self-reported depressive symptoms. Finally, analysis using multiple imputation (PROC MI/MIANALYZE) was performed for comparison with the complete case approach. Details are provided in eMethods 3.

Negative control outcomes were tested instead of depression to ensure the validity of the study design. History of cataract and hepatitis B were selected as non-psychiatric, non-respiratory negative control outcomes, where both perceived and biomarker-measured SHS were hypothesized to show nonsignificant associations with the outcomes. Meanwhile, history of asthma was selected as a non-psychiatric, but respiratory negative control outcome. In this analysis, biomarker-based SHS exposure was hypothesized to show a positive association with asthma, partially serving as a positive control for biomarker-based SHS exposure. However, perceived SHS was hypothesized to show nonsignificant associations with asthma, potentially acting as a negative control in the association with perceived SHS. Details are provided in eMethods 4.

Both analyses based on maximum likelihood estimation and the multiple imputation analysis require a 'missing at random' assumption to obtain unbiased results. Therefore, variables of the study were compared between complete cases and participants with at least one missing variable to assess whether it was plausible to attribute the missingness to the variables included. A detailed description is provided in eMethods 5. All analyses were conducted using SAS 9.4 (SAS Institute, Cary, NC).

### Ethical considerations

All procedures followed the ethical guidelines of the Declaration of Helsinki. The study received a waiver of informed consent due to the minimal risk associated with the study design (No. 4–2023-0420, Yonsei University Health System).

## Results

### Comparison of participant characteristics

Depressed and reference groups were compared in Table 1. Depressed participants were more likely to report perceived SHS exposure (37.47% vs 29.88%). The median urine cotinine concentrations were 0.48 (25th percentile: 0.35, 75th percentile: 1.05) ng/mL for non-depressed and 0.55 (25th percentile: 0.35, 75th percentile: 1.47) ng/mL for depressed participants, which resulted in depressed participants having significantly higher mean urine log-cotinine levels (0.12 vs −0.22). Additionally, depressed participants were more likely to be female compared to non-depressed participants. Depressed participants generally had lower socioeconomic status, were more frequently unmarried, and had fewer cohabitants. They also had a higher likelihood of being unemployed. Finally, depressed participants were more likely to report comorbidities.

**Table 1:** Characteristics of the study population by depression

	Total population	Not depressed (PHQ-9 < 10)	Depressed (PHQ-9 ≥ 10)	*p*-value^a^
	(*N* = 16,926)	(*N* = 16,064, 95.31%)	(*N* = 862, 4.69%)	
*Secondhand smoke exposure*				
Perceived secondhand smoke exposure				< 0.001
No	12,294 (69.76)	11,714 (70.12)	580 (62.53)	
Yes	4,632 (30.24)	4,350 (29.88)	282 (37.47)	
Log-cotinine^b^	−0.20 (1.76)	−0.22 (1.71)	0.12 (2.13)	< 0.001
* Demographic factors*				
Age	47.82 (16.65)	47.80 (16.53)	48.18 (18.82)	0.625
Sex				< 0.001
Male	5,979 (40.65)	5,809 (41.53)	170 (22.70)	
Female	10,947 (59.35)	10,255 (58.47)	692 (77.30)	
* Socioeconomic factors*				
Income^c^				< 0.001
Highest	4,406 (28.55)	4,305 (29.19)	101 (15.69)	
High	3,948 (25.51)	3,779 (25.68)	169 (21.92)	
Low	3,909 (23.66)	3,739 (23.78)	170 (21.38)	
Lowest	4,663 (22.28)	4,241 (21.35)	422 (41.01)	
Education				< 0.001
College or higher	6,173 (40.71)	5,977 (41.26)	196 (29.59)	
High school	5,405 (35.65)	5,159 (35.74)	246 (33.72)	
Middle school	1,749 (8.63)	1,649 (8.53)	100 (10.54)	
Elementary or lower	3,599 (15.02)	3,279 (14.47)	320 (26.14)	
Marital status				< 0.001
Married	14,274 (77.90)	13,579 (78.25)	695 (70.86)	
Unmarried	2,652 (22.10)	2,485 (21.75)	167 (29.14)	
Number of cohabitants	3.08 (0.02)	3.09 (0.02)	2.80 (0.06)	< 0.001
Occupation type				< 0.001
White-collar	6,220 (41.04)	6,017 (41.78)	203 (26.07)	
Blue-collar	3,508 (19.92)	3,393 (20.31)	115 (12.00)	
Unoccupied	7,198 (39.04)	6,654 (37.91)	544 (61.93)	
* Comorbidities*				
Hypertension				< 0.001
No	12,725 (80.27)	12,151 (80.61)	574 (73.45)	
Yes	4,201 (19.73)	3,913 (19.39)	288 (26.55)	
Diabetes				< 0.001
No	15,285 (92.36)	14,564 (92.64)	721 (86.67)	
Yes	1,641 (7.64)	1,500 (7.36)	141 (13.33)	
Dyslipidemia				< 0.001
No	13,659 (84.18)	13,041 (84.51)	618 (77.47)	
Yes	3,267 (15.82)	3,023 (15.49)	244 (22.53)	
Body mass index (kg/m^2^)				< 0.001
Underweight (< 18.5)	668 (4.30)	619 (4.14)	49 (7.67)	
Normal (18.5–23)	6,562 (38.99)	6,235 (39.07)	327 (37.27)	
Overweight (23–25)	3,914 (22.59)	3,750 (22.88)	164 (16.80)	
Obese (≥ 25)	5,782 (34.12)	5,460 (33.91)	322 (38.26)	
Cardiovascular disease				< 0.001
No	16,409 (97.83)	15,602 (97.98)	807 (94.79)	
Yes	517 (2.17)	462 (2.02)	55 (5.21)	
Neurovascular disease				< 0.001
No	16,536 (98.28)	15,727 (98.47)	809 (94.51)	
Yes	390 (1.72)	337 (1.53)	53 (5.49)	
Thyroid disease				< 0.001
No	16,177 (96.06)	15,376 (96.19)	801 (93.44)	
Yes	749 (3.94)	688 (3.81)	61 (6.56)	
Neoplastic disease				0.005
No	15,966 (95.27)	15,171 (95.38)	795 (93.11)	
Yes	960 (4.73)	893 (4.62)	67 (6.89)	

*Abbreviations* PHQ-9, Patient Health Questionnaire-9

Continuous variables are presented as mean (weighted sample standard deviation). Categorical variables (all other variables) are presented as N (weighted %)

^a^Continuous variables were tested using t-tests, and categorical variables with chi-square tests

^b^Cotinine (ng/mL) was log-transformed for statistical analyses

^c^Income was divided into survey weight adjusted quartiles in each survey year

### Association between perceived secondhand smoke exposure and depression

**Fig. 1:**
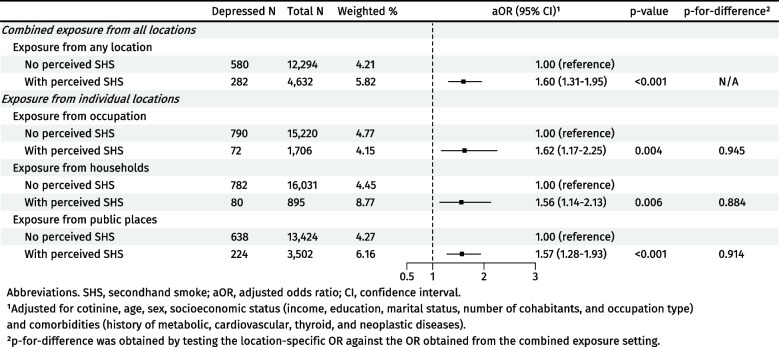
Odds ratios of depression associated with secondhand smoke (*N* = 16,926)

After adjusting for cotinine and other covariates, the association between perceived SHS and depression was significant (OR: 1.60, 95% CI: 1.31–1.95) (Fig. 1). The interaction between perceived and biomarker-based SHS was not significant (*p* = 0.541). Additionally, biomarker-measured SHS was also associated with depression, where a 1-unit increase in log-transformed cotinine was associated with increased odds of depression (OR: 1.11, 95% CI: 1.06–1.16) in the adjusted model.

Perceived SHS exposure from occupational (OR: 1.62, 95% CI: 1.17–2.25), household (OR: 1.56, 95% CI: 1.14–2.13), and public (OR: 1.57, 95% CI: 1.28–1.93) settings was associated with depression (Fig. 1). The strengths of these associations were similar to those in the original analysis (p-for-difference > 0.8 for all locations).

**Fig. 2:**
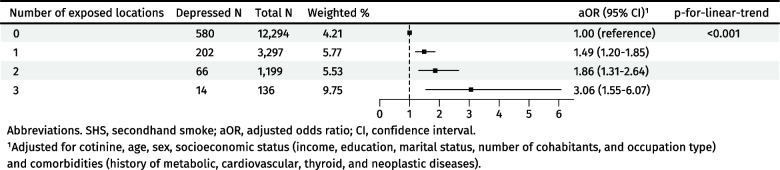
Dose–response association between number of locations of perceived SHS exposure and depression

The number of locations of perceived SHS exposure showed a dose–response relationship with depression (p-for-linear-trend < 0.001) (Fig. 2). Compared to perceived SHS exposure from a single location (OR: 1.49, 95% CI: 1.20–1.85), exposure from two locations (OR: 1.86, 95% CI: 1.31–2.64) and three locations (OR: 3.06, 95% CI: 1.55–6.07) showed stronger associations with depression.

### Effect heterogeneity analysis

Results of effect heterogeneity analyses are shown in eResults 1. Adding multiplicative interaction terms was significant when stratified by income (p-for-interaction = 0.008) (eResults 1). No significant heterogeneity was found for types of occupation (*p* = 0.178) or sex (*p* = 0.613) (eResults 1). The highest income group showed the strongest association between perceived SHS and depression, with an OR of 3.31 (95% CI: 2.03–5.39). Regarding additive interaction, high (RERI, Relative Excess Risk due to Interaction: −1.61), low (RERI: −1.23), and lowest (RERI: −1.61) income groups showed negative additive interaction.

### Sensitivity and negative control analyses

Results of sensitivity analyses are shown in eResults 2. Compared to the main analysis, all sensitivity analyses showed similar strengths of association and were statistically significant (eResults 2). Results of negative control analyses are displayed in eResults 3. Both non-psychiatric, non-respiratory negative control outcomes showed nonsignificant associations with perceived and biomarker-measured SHS exposure (eResults 3). The non-psychiatric, respiratory negative control outcome showed significant associations only with biomarker-measured exposure (eResults 3).

### Comparison between complete cases and cases with missing data

Participant characteristics were compared between complete cases and cases with at least one missing variable in eResults 4. Cases with missing variables reported depression more frequently. Additionally, these cases tended to be older and were more likely to be female. Furthermore, cases with missing variables showed lower socioeconomic status. Metabolic comorbidities such as hypertension and diabetes were more prevalent in cases with missing variables, while histories of cardiovascular, thyroid, and neoplastic diseases were more prevalent in complete cases.

## Discussion

This study found that perceived exposure to SHS was associated with depression, even after adjusting for biomarker-measured SHS exposure. This association was similar regardless of the location of exposure. Additionally, the number of locations exposed showed a positive dose–response association with depression.

To our knowledge, this is the first study to simultaneously evaluate perceived and biomarker-based SHS exposure in association with depression. Consistent with our findings, previous studies suggest that SHS and depression are associated [13], which has also been observed in the Korean population [34]. However, this study extends our understanding of the SHS-depression association by distinguishing between perceived and biological aspects of SHS exposure.

Simultaneous evaluation of both perceived and biomarker-based SHS exposure allowed comparison between the two types in their association with depression. Since perceived and biomarker-based exposures have different units, we calculated the increase in biomarker-measured exposure needed to show an association equivalent in strength to the perceived exposure-depression association. The strength of association that perceived exposure shows with depression is equivalent to a 4.45 increase in log-transformed cotinine, which translates into an 86-fold increase in urine cotinine levels. Therefore, to obtain the same strength of association as with perceived exposure, a hypothetical participant with median level exposure would have to be exposed to tobacco smoke at approximately twice the cut-off level (20.9 ng/mL) for discriminating non-smokers from smokers [35]. This highlights the importance of perceived SHS exposure over biomarker-measured exposure in the association with depression, since cases where non-smoking individuals are exposed to such high concentrations of tobacco smoke would be rare.

It is important to note that'perceived exposure'to SHS likely represents the detection of actual exposure at low levels, rather than purely subjective perception without any exposure. First, prior research on SHS perception suggests that individuals can detect SHS even at very low concentrations [36]. In addition, exposure to SHS typically occurs in brief and intermittent episodes, which commonly results in low urinary cotinine concentrations. Consistent with the descriptive statistics of our study, another study from Korea which used questionnaires adapted from the KNHANES reported that 36% of individuals reported experiencing SHS exposure at home with a mean exposure duration above 1.5 h per week, while the geometric mean of urine cotinine was approximately 0.5 ng/mL [37]. Another nationally representative study from Korea reported a geometric mean of urine cotinine of 2.1 ng/mL for non-smokers living with smokers [38]. Therefore, perceived exposure should be interpreted as detecting actual exposure that occurs at potentially low concentrations, rather than as perception without exposure.

The importance of perceived exposure over biological exposure on psychosomatic symptoms has been frequently emphasized in studies of environmental exposures. Air pollution, sharing similarities with SHS as hazardous substances involuntarily transmitted through air, is an example. A study restricted to alpine areas with limited air pollution found that perceived traffic air pollution was associated with somatic symptoms in adults and respiratory symptoms in children [39]. Another study using geospatially-modelled particulate matter concentrations suggests that perceived exposure, not modelled biological exposure, was associated with increased health risk perception, which in turn increased psychosomatic symptoms [40]. The emphasis on perceived exposure is not limited to air pollution studies. Perceived exposure to radiofrequency electromagnetic fields was associated with somatic symptoms and sleep disturbance after adjustment for modelled exposure dose [41]. Additionally, perceived exposure to herbicides was associated with post-traumatic stress symptoms and illicit drug use in Vietnam veterans after adjusting for combat experience [42]. In summary, the epidemiological evidence highlights the importance of perceived exposure on psychosomatic symptoms, which could also be applied to SHS exposure.

Exposure to SHS is overall a stressful event, which could increase the risk of depression. Acute exposure to SHS is considered an irritating and an annoying experience due to its odor and irritability to respiratory mucous membranes [17, 18]. Also, people consider exposure to SHS as harmful [17, 19], as its health consequences are well-known. Such aversive properties of SHS lead to reactions such as moving away or asking the person smoking not to smoke [43]. However, attempts to reduce exposure to SHS could be difficult when regulations are absent or the population is dense [44]. Moreover, individuals could be reluctant to confront smokers, while such negotiation attempts are frequently unsuccessful [44, 45], additionally contributing to SHS being experienced as a stressful life event. Given the evidence for the causal relationship between stressful life events and depression [46, 47], the stress due to exposure to SHS could contribute to increased depressive symptoms in the current study.

Another mechanism by which perceived SHS exposure could lead to depression might be explained by risk perception of SHS induced by exposure. The effect of risk perception on psychosomatic outcomes has been demonstrated by human experiments where negatively biased information was given about non-hazardous exposures. An experiment with an odorous chemical showed that participants who were given negatively biased information about the substance perceived the odor to be stronger and scored poorer on cognitive tasks [48]. Another experiment with sub-audible windfarm sounds also reported increased physical symptoms and worsened mood in participants who were given negatively biased information about the exposure [49]. Therefore, risk perception of SHS as a known cause of illnesses could lead to depression.

There are several strengths in the current study. Validated questionnaires and measures for exposure and outcome assessment were used on a large and representative sample of the Korean population. The association showed consistency across multiple exposure locations, while the number of locations of perceived exposure also demonstrated a dose–response relationship with depression. Furthermore, the study was able to suggest plausible mechanisms for the perceived SHS-depression association, and draw analogies to other environmental exposures. In addition, the study showed that the perceived SHS exposure-depression association was specific to mental health outcomes, based on the negative control analysis. Finally, by simultaneously evaluating both perceived and biomarker-based SHS exposure, the study demonstrated a relatively stronger association between perceived SHS exposure and depression compared to the association measured with biomarker-based exposure.

Despite these strengths, there are some limitations that could be addressed in future studies. First, the cross-sectional study design limits causal inference. Depression could increase perceived SHS exposure through increased passivity against SHS or increased sensitivity due to lower tolerability of stressors. However, results were consistent when participants with no prior physician diagnosis of depression were studied, reducing the possibility of reverse causality. Furthermore, due to the short half-life of cotinine, the biological SHS exposure during the 7-day assessment window of perceived SHS exposure may not have been fully captured. However, sensitivity analysis using NNAL, a biomarker with a longer half-life of several days that can account for a longer detection window, yielded consistent results. Another possible limitation is that the presence of hidden smokers could have affected the results. However, the findings were consistent after filtering out potential hidden smokers using biomarkers. Exclusion of participants with missing data could have biased the results. However, multiple imputation analyses were consistent with the original analysis, and comparison between complete cases and those with missing variables showed that the missingness could be attributed to the measured variables. Finally, other exposure-related covariates such as air pollution, and outcome-related covariates such as past mental health conditions and medications could not be evaluated from the KNHANES data.

There are also several additional questions that are worth evaluating in future research. First, the association between perceived SHS and depression could be heterogeneous across different sources of SHS. For example, heterogeneities may exist between SHS from electronic nicotine delivery systems (i.e., electronic cigarettes) versus traditional tobacco products, or between SHS from family members versus strangers. In addition, although this study evaluated dose–response relationships between the number of locations of perceived SHS exposure and depression, measurements of the actual severity and duration of perceived exposure should be both validated and assessed. Furthermore, this study focused on the association within non-smokers and ex-smokers. Therefore, SHS exposure among smokers, such as within designated smoking areas, is a necessary area for further research. Finally, although outdoor exposure may be prevalent, short, and temporary, the role of outdoor SHS exposure should also be evaluated.

## Conclusion

Perceived exposure to SHS was associated with depression independent of biomarker-measured exposure. The strength of association with depression was greater for perceived exposure compared to biomarker-measured exposure, suggesting a potentially significant association between perceived SHS exposure and depression even at minimal SHS concentrations. To minimize the exposure to SHS, extending the range of smokefree locations to locations such as individual households and small workplaces, overlooked by the current National Health Promotion Act of Korea, may be necessary. In cases of locations such as public places where indoor smoking is already banned, effective implementation of smoking bans, as well as monitoring for violations is necessary. In addition to essential countermeasures to reduce actual SHS exposure, measures to reduce cues of tobacco smoke, such as use of opaque smoking rooms to block visual cues, as well as frequent ventilation to limit olfactory cues, could be considered if smoking bans are not yet feasible. Creating a smokefree environment and thereby limiting perceived exposure to SHS may be necessary to protect the general population from depression.

## Supplementary Information

Supplementary Material 1.

## Data Availability

The original data that was used for analysis (Korea National Health and Nutrition Examination Survey) is available in the official website of the Ministry of Health of South Korea (URL: https://knhanes.kdca.go.kr/knhanes/main.do).

## Abbreviations

SHS: Secondhand smoke
KNHANES: Korea National Health and Nutrition Examination Survey
PHQ-9: Patient Health Questionnaire 9
OR: Odds ratio
CI: Confidence interval
RERI: Relative Excess Risk due to Interaction
NNAL: 4-(Methylnitrosamino)-1-(3-pyridyl)-1-butanol
