# Supplementary material for: Association between perceived exposure to secondhand smoke and depression independent of biomarker-measured exposure
Source: BMC Public Health. 2025 Aug 25;25:2917. doi: 10.1186/s12889-025-23967-8 (PMC12376406; doi:10.1186/s12889-025-23967-8)
Supplement: Supplementary file 1 — Supplementary Material 1. [file 12889_2025_23967_MOESM1_ESM.docx]

**eMethods 1. Definition of covariates.**

Age was treated as a continuous variable. Biological sex was used as a binary variable.

Income level was categorized into four groups (i.e., lowest, low, high, highest) based on weighted quartiles of monthly income for each survey year. Education level was categorized into four groups (i.e., elementary or lower, middle school, high school, college or higher) according to participants’ final academic achievements. Marital status (i.e., whether the participant had ever married) was treated as binary, and the number of cohabitants was used as a continuous variable. Occupation type was coded as a categorical variable with three categories (i.e., white-collar, blue-collar, unoccupied), defined by the individual’s current type of occupation. Participants who were employed but but temporarily resting were classified based on their original occupation type. Metabolic comorbidities were defined based on individuals’ history of hypertension, diabetes, dyslipidemia, and obesity, as indicated by body mass index (BMI, kg/m^2^). Cardiovascular disease was defined as history of angina pectoris or myocardial infarction. Neurovascular disease was defined by a history of ischemic or hemorrhagic stroke. Also, history of thyroid diseases was also included. Lastly, neoplastic disease was defined by a history of any form of malignancy. All comorbidities were binary, except for BMI, which was categorized into four groups (i.e., underweight, BMI<18.5; normal, 18.5≤BMI<23; overweight, 23≤BMI<25; obese, BMI≥25) according to the World Health Organization's BMI criteria for Asians [1].

**eMethods 2. Effect heterogeneity analysis**

Effect heterogeneity analysis was conducted based on income levels and occupation types, as the prevalence and perception of smoking vary by socioeconomic status and occupational factors [2]. Additionally, since females are generally at higher risk for mental health outcomes,

effect heterogeneity was tested by sex. Likelihood ratio tests were used to assess multiplicative interaction terms, with the significance level was set at 0.05/3 based on the Bonferroni correction. Relative Excess Risk due to Interaction (RERI) was also calculated in cases where statistically significant multiplicative interaction terms were found.

**eMethods 3. Sensitivity analysis**

First, the KNHANES dataset codes participants with age over 80 years old as 80, and participants with more than 6 cohabitants as 6. Since this loss of information could lead to residual confounding, age and the number of cohabitants were treated as categorical variables. Age was classified into 7 categories (ages 19 to 29, 30 to 39, 40 to 49, 50 to 59, 60 to 69, 70 to 79, 80 or older), and the number of cohabitants was classified into 6 categories (1 [living alone], 2, 3, 4, 5, 6 or more cohabitants).

Additionally, due to the cross-sectional nature of this study, depression could inversely alter the perception of secondhand smoke. Therefore, the analysis was restricted to those without prior physician-diagnosed depression to reduce the possibility of reverse causality.

Next, exposure dose adjustment based on biomarkers was conducted using 4-(methylnitrosamino)-1-(3-pyridyl)-1-butanol (NNAL, units: pg/mL) instead of cotinine to reflect a longer exposure period to tobacco smoke. A possible limitation of cotinine is its short half-life of 15 hours [3], which limits its ability to reflect long-term exposure to tobacco smoke [4]. Therefore, an alternative biomarker NNAL, with a half-life of 10-16 days [4], allows researchers to detect tobacco smoke exposure in the recent 6 to 12 weeks [5]. NNAL was experimentally measured from urine samples from a random subsample of participants in 2016 and 2018. The LOD for NNAL was 0.1006 pg/mL [6].

Furthermore, urine biomarkers were used to re-classify current smoking status. Misclassification of smoking history due to under-reporting of active smoking has been suggested [7], especially among Korean women, where up to 59% of biomarker-verified smokers were hidden smokers (i.e., those who self-reported as non-smokers) [8]. Based on the two available biomarkers, cotinine and NNAL, participants with cotinine>20.9 ng/mL or NNAL>8.9 pg/mL were re-classified as current smokers and removed from the analytic dataset [9].

In addition, alternative outcome definitions were tested. First, different cut-off scores for the PHQ-9 were used. Since the PHQ-9 can also assess depression of varying severities, a total score of 5 or above was used to examine mild depression, and 15 or above to examine moderately severe depression. Moreover, since the dataset for our study is limited to years when the PHQ-9 was applied, self-reported depressive symptoms lasting over 2 weeks in the past year were used as an alternative outcome variable in 2015, 2017, 2019, and 2021 to extend our analysis to larger populations (N=34,517). To match the outcome definitions above, the second question of the PHQ-9 ("Over the last 2 weeks, how often have you been bothered by feeling down, depressed, or hopeless?") was used for the years 2014, 2016, 2018, and 2020 to identify self-reported depressive symptoms. Participants who answered ‘not at all’ were classified as the reference group, while others were classified as experiencing depressive symptoms. In addition, we also tested whether both the years 2014, 2016, 2018, and 2020 and years 2015, 2017, 2019, and 2021 showed similar results with the original analysis separately.

Finally, since the original analysis is based on a complete case analysis (N=16,926), multiple imputation (PROC MI) was used to obtain a potentially less biased association. The imputation process estimated the missing variables for the adult non- and ex-smoker population (N=19,046). A fully conditional specification method for imputation was employed while accounting for the complex survey structure by using survey weights and combined strata-cluster as variables [10-12]. All variables included in the main analysis were also utilized in the model. Imputation was performed in order from the least (i.e., perceived secondhand smoke, cases with missing N=7) to the most (i.e., depression, cases with missing N=1,102) frequently missing variable. Continuous variables were imputed using the predictive mean matching linear regression method, binary and ordinal variables with the logistic regression method, and nominal variables with the discriminant function method [10-12]. Regression results from 25 imputed datasets were combined (PROC MIANALYZE) to estimate the association between perceived secondhand smoke and depression. A randomly chosen fixed seed was used to ensure the replicability of the study.

**eMethods 4. Negative control analysis**

Two types of negative control outcomes were selected to ensure the validity of the study design. This approach was taken because both the psychological effects due to the perception of secondhand smoke exposure and the direct biological effects of secondhand smoke are proposed as harmful effects of secondhand smoke. The first type of negative control outcome consists of non-psychiatric, non-respiratory outcomes with a clear cause of the outcome other than tobacco smoke. The second type of negative control outcome includes of non-psychiatric, respiratory outcomes that are known to be affected by tobacco smoke, but not through a psychiatric pathway. The association with perceived secondhand smoke was expected to be nonsignificant in both types of negative controls, while biomarker-measured exposure to secondhand smoke was expected to be significant in the non-psychiatric, respiratory negative controls.

Cataract and hepatitis B were selected as non-psychiatric, non-respiratory negative control outcomes. Cataracts are caused by aging, while trauma or radiation can also be possible causes. Additionally, hepatitis B is transmitted through direct bloodstream contact with the virus or contact with other body fluids. Both outcomes have known, clear causes other than tobacco smoke. Moreover, there is a lack of evidence for an association between the selected negative control outcomes and secondhand smoke. Participants with a current history of cataracts or positive testing for the hepatitis B surface antigen were classified as having the negative control outcome, while other participants were categorized as the reference group.

Asthma was selected as the non-psychiatric, respiratory negative control outcome. Asthma is caused by hyperreactivity of the airway, with known triggers including allergens, occupational chemicals, respiratory infections, and secondhand smoke [13]. Since the association between exposure to secondhand smoke and asthma may be attributed to the biochemical effects of secondhand smoke, biomarker-based secondhand smoke exposure was hypothesized to be associated with asthma, while perceived secondhand smoke exposure was hypothesized to show nonsignificant associations with asthma. Participants with a current history of asthma were classified as having the negative control outcome, while other participants were categorized as the reference group.

**eMethods 5. Comparison between complete cases and cases with missing data**

Both the main analysis (based on maximum likelihood estimation) and the multiple imputation analysis require a 'missing at random' (MAR) condition to obtain unbiased results. Therefore, the characteristics of the variables included in the study were compared between complete cases and participants with at least one missing variable. The number of participants in the group with missing variables differed for each comparison. For example, a case with any missing variable that also has the variable ‘depression’ missing cannot be used when comparing complete cases and cases with missing variables for ‘depression’. Such comparisons could help explain the missingness of variables based on other measured variables (e.g., females tend to not report their BMI) and test whether a MAR condition could be plausible. In contrast, a 'not missing at random' condition, which implies that the missingness is a result of the missing variables themselves (e.g., participants with high BMI tend to not report their BMI) cannot be statistically tested.

| **eResults 1. Odds ratios of depression associated with perceived secondhand smoke across stratification variables** | | | | | | |
| --- | --- | --- | --- | --- | --- | --- |
|  | No perceived SHS (N=12,294) | |  | With perceived SHS (N=4,632) | | p-for-interaction |
|  | N (%)^1^ | aOR (95% CI)^2^ |  | N (%)^1^ | aOR (95% CI)^2^ |  |
| Income |  |  |  |  |  | 0.008 |
| Highest | 47/3,136 (1.54) | 1.00 (reference) |  | 54/1,270 (4.88) | 3.31 (2.03-5.39) |  |
| High | 109/2,726 (3.71) | 2.35 (1.58-3.49) |  | 60/1,222 (4.72) | 3.05 (1.91-4.88) |  |
| Low | 102/2,729 (3.63) | 2.02 (1.34-3.05) |  | 68/1,180 (5.51) | 3.10 (1.98-4.86) |  |
| Lowest | 322/3,703 (8.38) | 3.86 (2.60-5.75) |  | 100/960 (9.46) | 4.99 (3.24-7.69) |  |
| Occupation type |  |  |  |  |  | 0.178 |
| White-collar | 101/4,196 (2.33) | 1.00 (reference) |  | 102/2,024 (4.26) | 1.65 (1.20-2.28) |  |
| Blue-collar | 79/2,382 (2.73) | 1.00 (0.71-1.43) |  | 36/1,126 (3.00) | 1.23 (0.77-1.95) | - |
| Unoccupied | 400/5,716 (6.56) | 1.96 (1.50-2.57) |  | 144/1,482 (10.31) | 3.28 (2.37-4.54) | - |
| Sex |  |  |  |  |  | 0.613 |
| Male | 113/4,099 (2.29) | 1.00 (reference) |  | 57/1,880 (3.25) | 1.51 (1.00-2.27) |  |
| Female | 467/8,195 (5.38) | 2.30 (1.77-2.98) |  | 225/2,752 (8.08) | 3.75 (2.82-4.98) |  |
| *Abbreviations*. SHS, secondhand smoke; aOR, adjusted odds ratio; CI, confidence interval; RERI, relative excess risk due to interaction. | | | | | | |
| ^1^N (%) displays the strata-specific depressed N/total N (weighted %). | | | | | | |
| ^2^All models were adjusted for cotinine, age, sex, socioeconomic status (income, education, marital status, number of cohabitants, and occupation type) and comorbidities (history of metabolic, cardiovascular, thyroid, and neoplastic diseases), except for the stratification variable. | | | | | | |


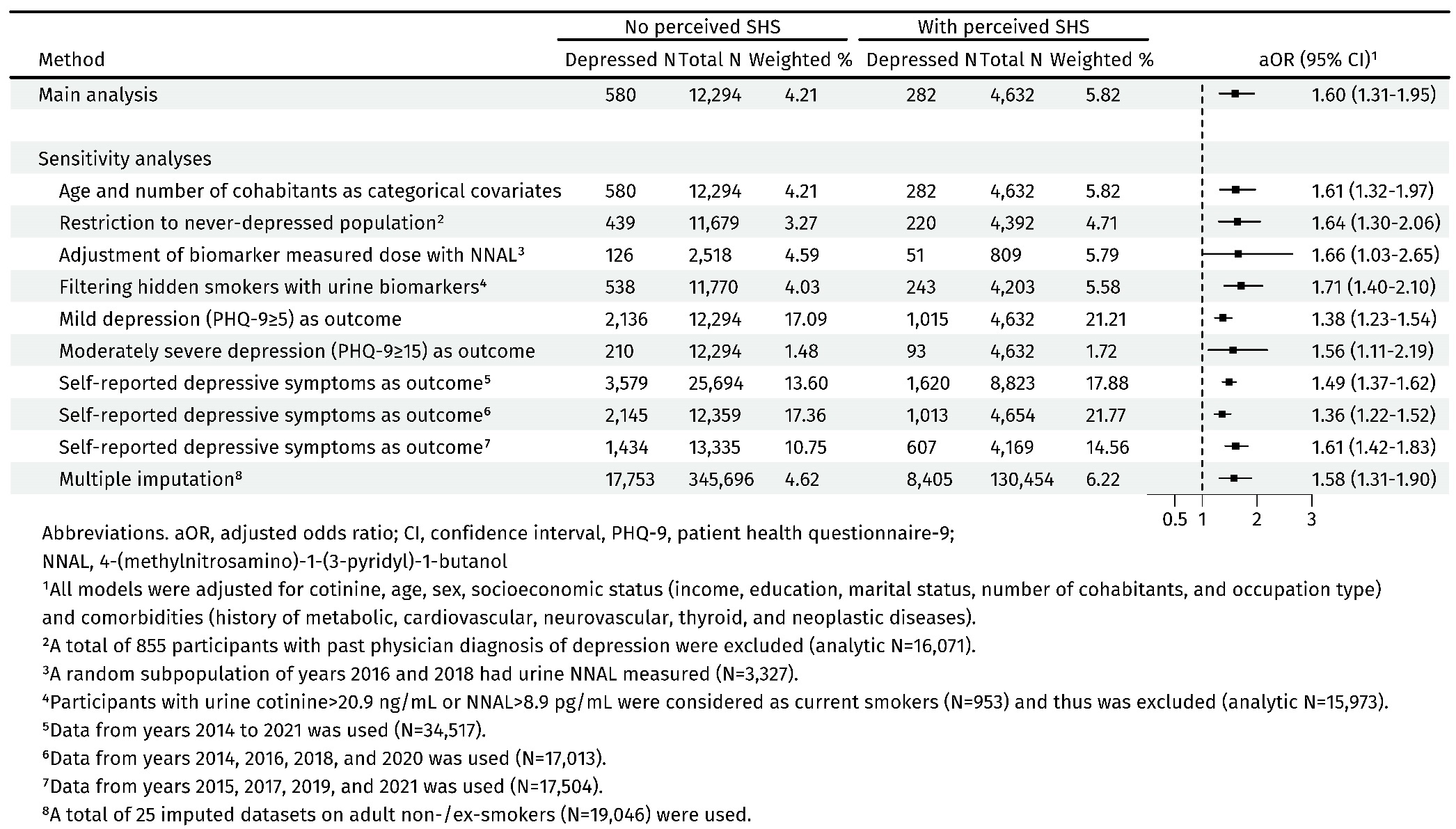


**eResults 2. Summary of sensitivity analyses**

| **eResults 3. Odds ratios of negative control outcomes associated with perceived secondhand smoke** | | | | | | | | | | | |
| --- | --- | --- | --- | --- | --- | --- | --- | --- | --- | --- | --- |
|  | No perceived SHS | | |  | With perceived SHS | | |  | Perceived SHS |  | Biomarker measured SHS^1^ |
|  |  |  |  |  |  |  |  |  |  |  |  |
|  | Depressed N | Total N | Weighted % |  | Depressed N | Total N | Weighted % |  | aOR (95% CI)^2^ | | |
|  |  |  |  |  |  |  |  |  |  |  |  |
| Non-psychiatric, non-respiratory negative control outcome | | | | | | | | | | | |
| Cataract | 520 | 12,294 | 2.93 |  | 96 | 4,632 | 1.48 |  | 1.07 (0.78-1.47) |  | 0.95 (0.86-1.05) |
| Hepatitis B | 348 | 12,294 | 2.91 |  | 153 | 4,632 | 3.39 |  | 1.16 (0.91-1.47) |  | 1.00 (0.94-1.06) |
| Non-psychiatric, respiratory negative control outcome | | | | | | | | | | | |
| Asthma | 243 | 12,294 | 1.74 |  | 71 | 4,632 | 1.42 |  | 0.97 (0.69-1.35) |  | 1.09 (1.01-1.18) |
| *Abbreviations.* SHS, secondhand smoke; aOR, adjusted odds ratio; CI, confidence interval. | | | | | | | | | | | |
| ^1^The OR was obtained for 1 unit of increase in log-transformed cotinine. | | | | | | | | | | | |
| ^2^All models were adjusted for cotinine, age, sex, socioeconomic status (income, education, marital status, number of cohabitants, and occupation type) and comorbidities (history of metabolic, cardiovascular, thyroid, and neoplastic diseases). | | | | | | | | | | | |

| **eResults 4. Comparison between complete cases and cases with missing variables** | | | |
| --- | --- | --- | --- |
|  | Complete | Cases with | p-value^1^ |
|  | cases | missing variables |  |
| *Outcome variable* |  |  |  |
| Depression |  |  | 0.045 |
| No (PHQ-9<10) | 16,064 (95.31) | 954 (93.71) |  |
| Yes (PHQ-9≥10) | 862 (4.69) | 64 (6.29) |  |
| *Secondhand smoke exposure* |  |  |  |
| Perceived secondhand smoke exposure | |  | 0.335 |
| No | 12,294 (69.76) | 1,530 (68.47) |  |
| Yes | 4,632 (30.24) | 584 (31.53) |  |
| Cotinine (ng/mL)^2^ | 0.48 (0.71) | 0.39 (0.65) | 0.924 |
| *Demographic factors* |  |  |  |
| Age | 47.82 (0.23) | 50.57 (0.64) | <0.001 |
| Sex |  |  | <0.001 |
| Male | 5,979 (40.65) | 594 (30.23) |  |
| Female | 10,947 (59.35) | 1,526 (69.77) |  |
| *Socioeconomic factors* |  |  |  |
| Income^3^ |  |  | <0.001 |
| Highest | 4,406 (28.55) | 393 (21.14) |  |
| High | 3,948 (25.51) | 413 (23.66) |  |
| Low | 3,909 (23.66) | 452 (24.84) |  |
| Lowest | 4,663 (22.28) | 798 (30.36) |  |
| Education |  |  | <0.001 |
| College or higher | 6,173 (40.71) | 358 (34.75) |  |
| High school | 5,405 (35.65) | 342 (33.42) |  |
| Middle school | 1,749 (8.63) | 81 (7.08) |  |
| Elementary or lower | 3,599 (15.02) | 390 (24.74) |  |
| Marital status |  |  | 0.219 |
| Married | 14,274 (77.90) | 1,806 (79.55) |  |
| Unmarried | 2,652 (22.10) | 314 (20.45) |  |
| Number of cohabitants | 3.08 (0.02) | 2.98 (0.04) | 0.008 |
| Occupation type |  |  | <0.001 |
| White-collar | 6,220 (41.04) | 346 (34.19) |  |
| Blue-collar | 3,508 (19.92) | 168 (13.53) |  |
| Unoccupied | 7,198 (39.04) | 644 (52.28) |  |
| *Comorbidities* |  |  |  |
| Hypertension |  |  | 0.004 |
| No | 12,725 (80.27) | 1,502 (77.08) |  |
| Yes | 4,201 (19.73) | 618 (22.92) |  |
| Diabetes |  |  | 0.044 |
| No | 15,285 (92.36) | 1,873 (91.00) |  |
| Yes | 1,641 (7.64) | 247 (9.00) |  |
| Dyslipidemia |  |  | 0.090 |
| No | 13,659 (84.18) | 1,776 (85.90) |  |
| Yes | 3,267 (15.82) | 344 (14.10) |  |
| Body mass index (kg/m^2^) |  |  | 0.549 |
| Underweight (<18.5) | 668 (4.30) | 87 (4.42) |  |
| Normal (18.5-23) | 6,562 (38.99) | 819 (40.77) |  |
| Overweight (23-25) | 3,914 (22.59) | 435 (22.30) |  |
| Obese (≥25) | 5,782 (34.12) | 657 (32.51) |  |
| Cardiovascular disease |  |  | 0.047 |
| No | 16,409 (97.83) | 2,075 (98.48) |  |
| Yes | 517 (2.17) | 45 (1.52) |  |
| Neurovascular disease |  |  | 0.541 |
| No | 16,536 (98.28) | 2,068 (98.09) |  |
| Yes | 390 (1.72) | 52 (1.91) |  |
| Thyroid disease |  |  | <0.001 |
| No | 16,177 (96.06) | 2,079 (98.10) |  |
| Yes | 749 (3.94) | 41 (1.90) |  |
| Neoplastic disease |  |  | 0.008 |
| No | 15,966 (95.27) | 2,040 (96.65) |  |
| Yes | 960 (4.73) | 80 (3.35) |  |
| *Abbreviations*. PHQ-9, Patient Health Questionnaire-9. | | | |
| Age, and number of cohabitants are presented as mean (standard error). Cotinine is presented as median (interquartile range). Categorical variables (all other variables) are presented as N (weighted %). | | | |
| ^1^Continuous variables were tested using t-tests, and categorical variables with chi-square tests. | | | |
| ^2^Cotinine was log-transformed for statistical analyses. | | | |
| ^3^Income was divided into survey weight adjusted quartiles in each survey year. | | | |

**Reference**

1. World Health Organization: **The Asia-Pacific perspective: redefining obesity and its treatment**. Sydney: Health Communications Australia; 2000.

2. Hiscock R, Bauld L, Amos A, Fidler JA, Munafò M: **Socioeconomic status and smoking: a review**. *Annals of the New York Academy of Sciences* 2012, **1248**(1):107-123.

3. Jarvis MJ, Russell M, Benowitz NL, Feyerabend C: **Elimination of cotinine from body fluids: implications for noninvasive measurement of tobacco smoke exposure**. *American journal of public health* 1988, **78**(6):696-698.

4. Goniewicz ML, Eisner MD, Lazcano-Ponce E, Zielinska-Danch W, Koszowski B, Sobczak A, Havel C, Jacob P, Benowitz NL: **Comparison of urine cotinine and the tobacco-specific nitrosamine metabolite 4-(methylnitrosamino)-1-(3-pyridyl)-1-butanol (NNAL) and their ratio to discriminate active from passive smoking**. *Nicotine & Tobacco Research* 2011, **13**(3):202-208.

5. Goniewicz ML, Havel CM, Peng MW, Jacob III P, Dempsey D, Yu L, Zielinska-Danch W, Koszowski B, Czogala J, Sobczak A: **Elimination kinetics of the tobacco-specific biomarker and lung carcinogen 4-(methylnitrosamino)-1-(3-pyridyl)-1-butanol**. *Cancer epidemiology, biomarkers & prevention* 2009, **18**(12):3421-3425.

6. Lee H-S: **Diagnostic Performance Evaluation of the Novel Index Combining Urinary Cotinine and 4-(Methylnitrosamino)-1-(3-pyridyl)-1-butanol in Smoking Status Verification and Usefulness for Trend Monitoring of Tobacco Smoking Exposure**. *International Journal of Environmental Research and Public Health* 2022, **19**(19):12147.

7. Jung SJ, Shin A, Kang D: **Active smoking and exposure to secondhand smoke and their relationship to depressive symptoms in the Korea national health and nutrition examination survey (KNHANES)**. *BMC Public Health* 2015, **15**(1):1-11.

8. Jung-Chio K-H, Khang Y-H, Cho H-J: **Hidden female smokers in Asia: a comparison of self-reported with cotinine-verified smoking prevalence rates in representative national data from an Asian population**. *Tobacco control* 2012, **21**(6):536-542.

9. Park EY, Lim MK, Park E, Kim Y, Lee D, Oh K: **Optimum Urine Cotinine and NNAL Levels to Distinguish Smokers from Non-Smokers by the Changes in Tobacco Control Policy in Korea from 2008 to 2018**. *Nicotine & Tobacco Research* 2022, **24**(11):1821-1828.

10. He Y, Zhang G: **Multiple Imputation of Missing Complex Survey Data using SAS®: A Brief Overview and An Example Based on the Research and Development Survey (RANDS)**. 2023.

11. Liu Y, De A: **Multiple imputation by fully conditional specification for dealing with missing data in a large epidemiologic study**. *International journal of statistics in medical research* 2015, **4**(3):287.

12. Berglund P: **Multiple imputation using the fully conditional specification method: a comparison of SAS®, Stata, IVEware, and R**. *Proceedings of the SAS Global Forum 2015 Conference* 2015:2081-2015.

13. Gautier C, Charpin D: **Environmental triggers and avoidance in the management of asthma**. *Journal of asthma and allergy* 2017:47-56.
